# Deep learning radiomics nomogram predicts lymph node metastasis in laryngeal squamous cell carcinoma

**DOI:** 10.3389/fonc.2025.1573687

**Published:** 2025-08-12

**Authors:** Yun Liang, Min He, Wenqing Chen, Lizhen Li, Yumeng Dong, Gang Liang, Hui Huangfu, Zengyu Jiang, Sheng He

**Affiliations:** ^1^ Department of Radiology, First Hospital of Shanxi Medical University, Taiyuan, China; ^2^ College of Medical Imaging, Shanxi Medical University, Taiyuan, China; ^3^ Department of Pathology, First Hospital of Shanxi Medical University, Taiyuan, China; ^4^ Department of Otolaryngology-Head and Neck Surgery, First Hospital of Shanxi Medical University, Taiyuan, Shanxi, China; ^5^ Collaborative Innovation Center for Frontier Medicine, Shanxi Medical University-Tsinghua Medicine College, Taiyuan, Shanxi, China

**Keywords:** deep learning, artificial intelligence, radiomics, laryngeal cancer, lymph node metastasis

## Abstract

**Background:**

Lymph node metastases (LNM) in laryngeal squamous cell carcinoma (LSCC) has been associated with lower survival, but current imaging methods, such as computed tomography (CT), have limited capabilities to identify them. Both conventional radiomics, involving data analysis of high-throughput quantitative features extracted from medical images, as well as deep learning networks, improved LNM diagnostic accuracy in LSCC, but the combination of both approaches has not been fully examined. In this study, we aimed to improve LNM identification in LSCC patients by developing a predictive nomogram, combining deep learning radiomics and clinical imaging features from CT images.

**Methods:**

A retrospective analysis of 235 LSCC patients, divided into training (164) and validation (71) sets, was conducted. Radiomics features were extracted from CT images, and 7 machine learning algorithms were used to develop 7 radiomics models, which were combined with deep learning features extracted from the ResNet50 deep learning network to form deep learning radiomics (DLR) models. The optimal DLR model was combined with significant clinical imaging features from CT scans to develop the predictive nomogram for LNM in LSCC.

**Results:**

The nomogram, under receiver operating characteristic (ROC) curve analyses, yielded areas under the curve (AUC) values of, respectively, 0.934 and 0.864 for training and validation sets, significantly higher than clinical imaging features (0.832 and 0.817), conventional radiomics (0.861 and 0.818), and DLR (0.913 and 0.864), indicating that it was significantly more accurate in predicting LNM in LSCC patients. Additionally, decision curve analysis found that the nomogram had significantly higher clinical utility than the other 3 models.

**Conclusion:**

The predictive nomogram, combining clinical imaging and DLR features, is able to accurately identify LNM in LSCC patients, providing valuable information for non-invasive LN staging and personalized treatment approaches.

## 1 Introduction

Laryngeal cancer is one of the most common malignant tumors of the head and neck, of which laryngeal squamous cell carcinoma (LSCC) accounts for ~85-95% (1, 2). Its incidence in China is currently on the rise, with high mortality rates (3, 4). The presence of cervical lymph node metastases (LNM) in LSCC have been associated with decreased overall survival, and recurrence in regional LNs is a major cause behind LSCC treatment failures (5). As a result, accurate preoperative identification of LN statuses could aid in developing effective treatments for LSCC, as well as more precise patient prognostications. Currently, to detect primary tumors and regional LNM, a number of traditional non-invasive imaging methods, such as computed tomography (CT), magnetic resonance imaging (MRI), and positron emission tomography-CT, are widely used, but their ability to distinguish between metastatic and non-metastatic LNs are limited (6, 7). However, the application of radiomics, which involves data analysis of high-throughput quantitative features extracted from traditional medical images, is able to improve LNM diagnostic accuracy, in turn increasing the reliability of LSCC patient prognoses; this has led to it becoming increasingly important in cancer research (8, 9).

Wider applications of traditional radiomics, though, are still hindered by being relatively time-consuming, as well as their results being subjective in nature. This has consequently led to the development of “deep learning radiomics (DLR)”, an innovative method that is able to conduct end-to-end learning, as well as automatically discover specific predictive indices to improve the accuracy of prediction models (10, 11). Multiple studies have either used traditional radiomics or deep learning alone to predict LNM in LSCC (12–14), but few studies have been carried out to investigate the ability of DLR to predict LNM in LSCC. In this study, we aim to fill in this gap by investigating whether a DLR model, based on preoperative CT images, combined with other clinical imaging features, could accurately predict pre-surgical LN statuses for LSCC patients. We found that a combination of the clinical imaging features of LNM being reported in CT scans, as well as whether the primary LSCC tumor was located in the glottis or super-glottis, with the DLR model, using the XGBoost machine learning algorithm for radiomics, and the ResNet50 deep learning network, could serve as the basis for a predictive nomogram. The nomogram was significantly more accurate for predicting LNM in LSCC versus either clinical imaging features, conventional radiomics, or DLR alone, thereby providing a tool for guiding LSCC treatment.

## 2 Materials and methods

### 2.1 Patient recruitment, inclusion and exclusion criteria

**Figure 1:**
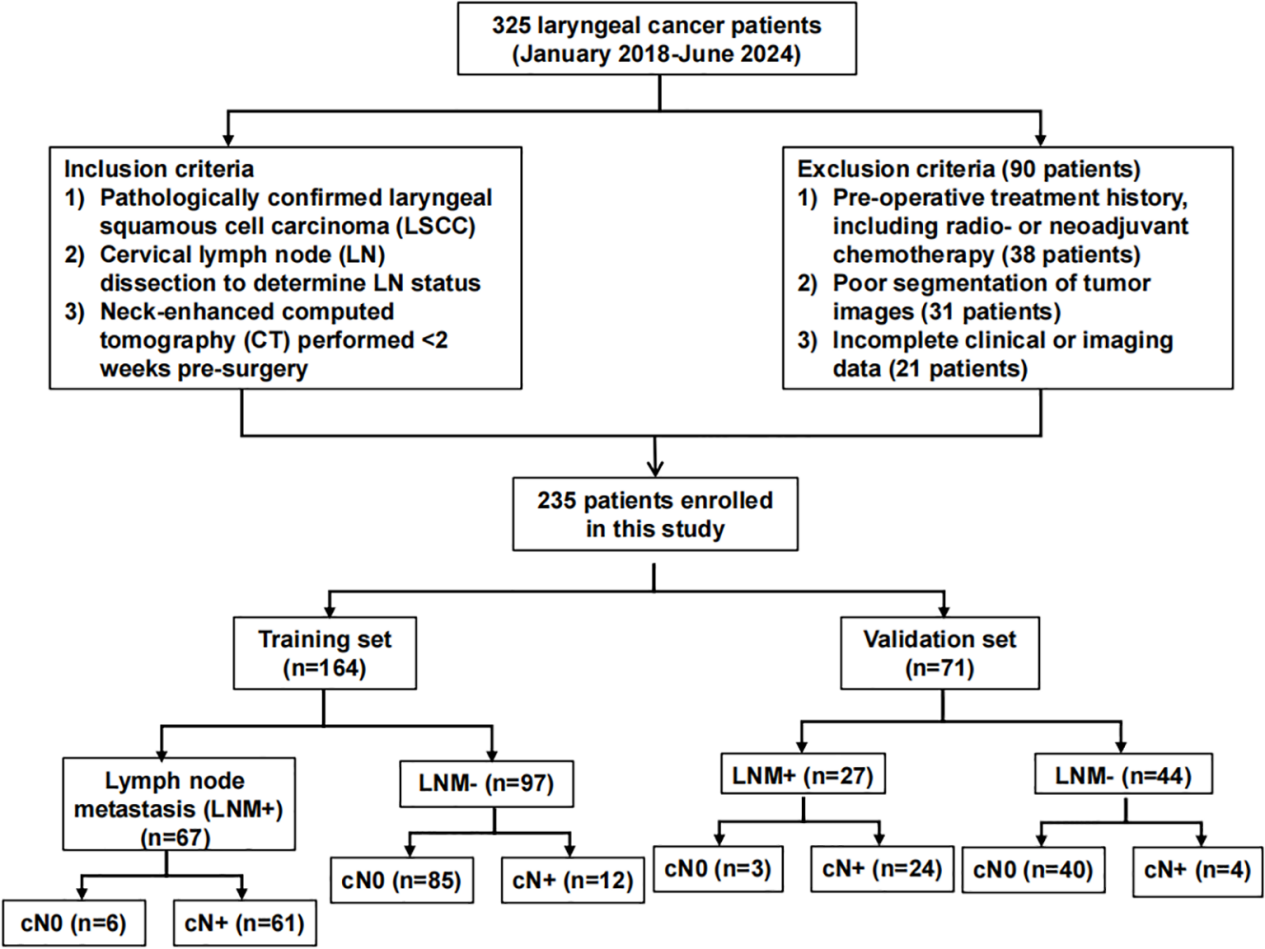
Flow chart of overall patient recruitment.

This study was a retrospective analysis of 235 patients, obtained after inclusion and exclusion criteria were applied, with pathologically confirmed LSCC, who underwent radical open surgery and neck LN dissection at the First Hospital of Shanxi Medical University, between January 2018-June 2024. Inclusion criteria were as follows: 1) Having pathologically confirmed LSCC, both non- (cN0) and metastatic (cN+), 2) LN statuses confirmed by neck LN dissection, or 3) Neck-enhanced CT performed <2 weeks pre-surgery, while exclusion criteria were: 1) Pre-operative treatment history, including radiotherapy or neoadjuvant chemotherapy, 2) Poor segmentation of tumor images, or 3) Incomplete clinical or imaging data. cN+ patients that were included in this study were diagnosed based on the imaging criteria defined by van den Brekel et al. (15); briefly, cN+ was present if at least 1 of the following conditions were met: 1) Short-axis LN diameter >1 cm, 2) ≥2 borderline LN, which was defined as having short-axis diameters >0.8 cm, in common drainage regions, or 3) Peripheral rim enhancement of LN. If any of those 3 conditions were not met, the patient was defined as having cN0. **Figure 1** shows the flow-chart of the overall patient recruitment process.

### 2.2 CT scanning and obtaining images

All patients underwent preoperative CT scanning, using multi-slice CT (GE 64-slice 128-slice Light Speed spiral CT, USA). Each patient was scanned, in a supine position, from the base of the skull to the lower edge of the sternoclavicular joint; they breathed quietly and had no swallowing. CT imaging parameters were: tube voltage 120 kV, tube current was automatically controlled, and image matrix 512×512, yielding CT “slice” thicknesses of 0.625 mm. During the CT scan, 1.5 ml/kg of intravenous contrast agent (UltraVision, 370 mg I/mL) was injected into the antecubital vein, at a rate of 3.0 mL/s, via an automatic power injector; this yielded a venous phase enhanced image 60–65 s after contrast agent injection. The resulting neck enhanced CT image (DICOM format) was then exported using the Picture Archiving and Communication System of the First Hospital of Shanxi Medical University.

### 2.3 Tumor image segmentation and quantitative feature extraction for radiomics

To achieve clear visualization of head and neck tissues, CT images were standardized, with window width at 350, level at 50, and Hounsfield unit values between -125–225. These images were then resampled to a voxel size of 1×1×1 mm with the bilinear interpolation method, and the tumor boundary was outlined independently by 2 experienced radiologists, using 3D-Slicer software (v5.6.1). The 2 radiologists (Dr. A and B) also referred to images obtained from trans-nasal fiberoptic examination of the larynx to assist in defining the tumor boundary and region of interest (ROI), as shown in **Supplementary Figure 1**. Intra-inter-class correlation coefficient (ICC) was used, based on based on Berenguer et al. (16), to evaluate the reproducibility of radiomics features extracted from tumor ROIs drawn by Dr. A and B. The ROI was confirmed by a 3^rd^ physician (Dr. C) with >10 years of experience in diagnosing head and neck diseases. More specifically, to evaluate the stability and reproducibility of the obtained radiomics features, as well as ensure inter-observational repeatability, tumor images from 30 patients were selected, and ROIs re-drawn 1 month later for feature extraction, by Dr. C. The overall study workflow is shown in **Supplementary Figure 2**.

Radiomics features were extracted using the PyRadiomics package, and 1403 were extracted, including first-order, shape-based, and textural features. For deep learning feature extraction, the image containing the largest tumor ROI was cropped and resized to a standardized size of 256×256 pixels, using a linear interpolation method. Considering the relatively small size of the cropped and resized image, data augmentation methods were then used, including random horizontal and vertical flipping, as well as cropping to a size of 224×224 pixels. The ResNet50 network was pre-trained using the ImageNet dataset, followed by transfer learning being performed on the training set. The parameters used in ResNet50 were iteratively updated via backpropagation, using a cross-entropy loss function; this function was computed between output probabilities and pathological labels. A 1×10^−4^ learning rate was set, and the Adam optimizer was utilized for parameter updates. To mitigate overfitting, a batch size of 64 was used, with L2 regularization and early stopping strategies being implemented. Additionally, data imputed into ResNet50 from pre-processed images were normalized using the default ImageNet mean-subtraction method. Model parameters were also fine-tuned, based on pre-trained model parameters derived from the training data. After training, the penultimate average pooling layer was used as the deep learning feature, for a total of 2048 deep learning features.

### 2.4 Feature screening and model building for predicting LNM in LSCC

All extracted features were normalized using Z-score, and their statistical significances were evaluated using Students t-test; only features with p<0.05 were retained. The correlation of those retained features with high repeatability was evaluated based on Spearman correlation coefficients, in which if any 2 features had correlation coefficients>0.9, 1 of those 2 features was retained. Afterwards, the optimal radiomics features were identified using the least absolute selection and shrinkage operator (LASSO) regression analysis, with 10-fold cross-validation (**Supplementary Figures 3A, B**). Radiomics features were fused with deep learning features using the early fusion method; the optimal deep learning features were screened out using the same methods as that of radiomics, namely Z-score, Students t-test, Spearman correlation coefficients, and LASSO with 10-fold cross-validation (**Supplementary Figure 3C**). Conventional radiomics and DLR models were built, using logistic regression (LR), support vector machine (SVM), random forest (RF), ExtraTrees, XGBoost, light gradient boosting machine (GBM), and feedforward neural network multilayer perceptron (MLP) machine learning algorithms. The optimal hyperparameter combinations for those algorithms were identified by the Grid Search method.

To construct the predictive nomogram model for LNM in LSCC, the most optimal radiomics model was combined with the most statistically significant clinical risk factors, which were identified by uni- and multivariate logistic regression analyses. The predictive accuracy of this model, along with that of clinical risk factors alone, radiomics, and DLR, were evaluated using receiver operating characteristic (ROC) curve analysis, followed by decision curve analyses (DCA) to examine the extent of their clinical utility.

### 2.5 Statistical analysis

The analyses were performed using Python (version 3.7.12) and Statsmodels (version 0.13.2). The development of our machine learning models utilized the Scikit-learn (version 1.0.2) interface. Deep learning training was conducted on an NVIDIA 4090 GPU, with MONAI 0.8.1 and PyTorch 1.8.1 frameworks.

Clinical imaging characteristics were confirmed to have either non- or normal distributions with the Shapiro-Wilk test, and continuous variables were analyzed with either Students t test, if they had a normal distribution, or Mann-Whitney U test, if they did not. Categorical variables were analyzed using the χ² test. P<0.05 was considered statistically significant.

## 3 Results

### 3.1 The clinical imaging characteristics model is predictive for LNM in LSCC

The 235 patients were randomly divided in a 7:3 ratio into training (164) and validation (71) sets. No significant differences in the clinical imaging characteristics of age, gender, the presence of LSCC metastases in CT scans, primary tumor location being in the glottis or supra-glottis, as well as whether LSCC was at stages T1–2 or 3-4, were present between the 2 sets (**Table 1**).

**Table 1:** Clinical imaging characteristics of laryngeal squamous cell carcinoma (LSCC) patients.

Characteristics	Training set (n=164)	Validation set (N=71)	χ^2^/t-value	p-value
Age (years)	63.45 ± 8.970	62.45 ± 9.085	0.777	0.438
Gender			0.133	0.716
Male	133	59		
Female	31	12		
Computed Tomography (CT) feature			0.521	0.470
Metastasis	73	28		
No Metastasis	91	43		
Primary tumor location			0.255	0.614
Glottis	59	28		
Supra-glottis	105	43		
T stage			0.571	0.450
T1-2	54	27		
T3-4	110	44		

The 235 patients were also divided into 2 groups, based on local LN statuses, in which those with positive LN (94 patients) were significantly more likely to also have LNM, and their tumors were more often located in the supra-glottis. By contrast, patients with negative LN were less likely to have LNM, but were more likely to be located in the glottis (**Table 2**). No significant differences were found for other clinical imaging characteristics (**Table 2**).

**Table 2:** Comparison of clinical imaging features based on different lymph node (LN) statuses.

Characteristics	Positive LN (n=94)	Negative LN (n=141)	χ^2^/t-value	p-value
Age (years)	62.53 ± 9.558	63.55 ± 8.613	0.852	0.395
Gender			0.171	0.679
Male	78	114		
Female	16	27		
CT Feature			59.178	<0.001
Metastasis	69	32		
No Metastasis	25	109		
Primary tumor location			63.075	<0.001
Glottis	6	81		
Supra-Glottis	88	60		
T stage			3.215	0.073
T1-2	26	55		
T3-4	68	86		

The associations between different clinical imaging characteristics with the absence or presence of LNM was then examined by uni- and multi-variate logistic regression analyses, in which it was found that CT reports of LNM, as well as primary tumor location, were significantly associated with actual LNM occurrence for LSCC (**Table 3**). Therefore, CT reports of LNM, as well as whether the primary tumor was in the glottis or supra-glottis, were incorporated into the “clinical imaging features model”, which was found under ROC curve analyses to be highly predictive for LNM in LSCC, with areas under the curve (AUC) of 0.832 (95% CI 0.775-0.889) and 0.817 (95% CI 0.727-0.908), for respectively, the training and validation sets.

**Table 3:** Uni- and multi-variate analyses between LN metastases (LNM) versus non-LNM groups for clinical imaging characteristics, expressed as odds ratio (OR) (95% confidence interval [CI]).

Characteristics	Univariate analysis OR (95% CI)	p-value	Multivariate analysis OR (95% CI)	p-value
Age	0.987 (0.959-1.017)	0.394		
Gender	1.155 (0.584-2.284)	0.680		
CT Feature	9.401 (5.140-17.196)	<0.001	0.210 (0.108-0.408)	<0.001
Primary Tumor Location	19.800 (8.116-48.303)	<0.001	0.092 (0.036-0.235)	<0.001
T Stage	1.673 (0.951-2.942)	0.074		

### 3.2 XGBoost DLR model was more highly predictive for LNM compared to other conventional and DLR models

**Figure 2:**
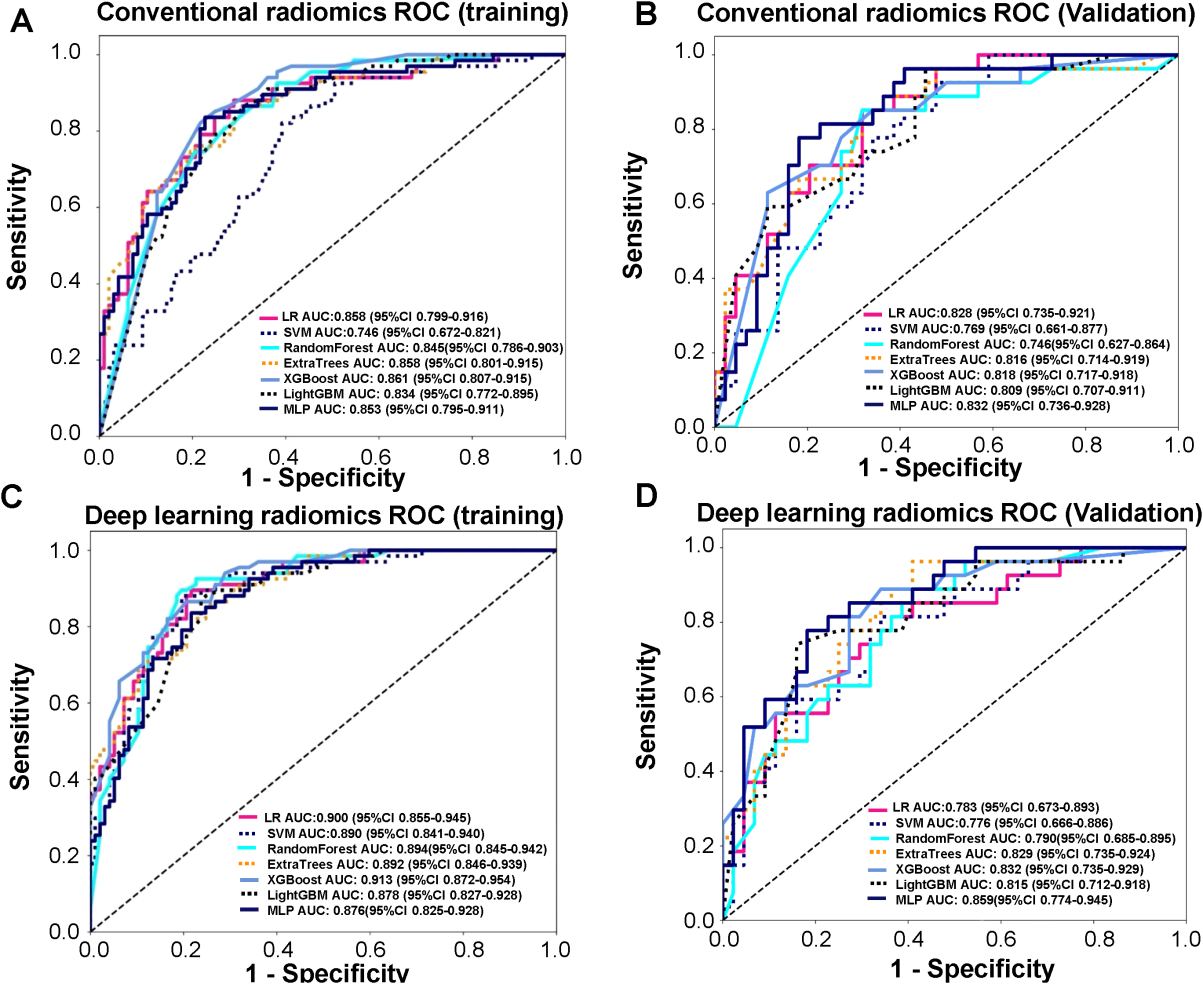
Receiver operating characteristic (ROC) curve analyses, conducted on the training set, for accurately predicting lymph node metastasis (LNM) in laryngeal squamous cell carcinoma (LSCC) patients. **(A)** ROC curves in training set for 7 conventional radiomics models, based on the machine learning algorithms of logistic regression (LR), support vector machine (SVM), random forest (RF), ExtraTrees, XGBoost, light gradient boosting machine (GBM), and feed-forward neural network multilayer perceptron (MLP), **(B)** ROC curves in validation for 7 conventional radiomics models, based on the machine learning algorithms of logistic regression (LR), support vector machine (SVM), random forest (RF), ExtraTrees, XGBoost, light gradient boosting machine (GBM), and feed-forward neural network multilayer perceptron (MLP), **(C)** ROC curves in training set for 7 deep learning radiomics (DLR) models, comprising of LR, SVM, RF, ExtraTrees, XGBoost, Light GBM, and MLP radiomics models combined with deep learning features, **(D)** ROC curves in validation set for 7 deep learning radiomics (DLR) models, comprising of LR, SVM, RF, ExtraTrees, XGBoost, Light GBM, and MLP radiomics models combined with deep learning features.

Based on conventional radiomics and deep learning radiomics analyses, a total of 15 radiomics, and 12 DLR features, were selected. For both types of features, 7 models each were constructed, based on the machine learning algorithms of LR, SVM, RF, ExtraTrees, XGBoost, light GBM, and MLP. We found that under ROC curve analyses, the XGBoost model, for both conventional radiomics and DLR, was the most strongly accurate for predicting LNM among LSCC patients. Indeed, AUC values for the XGBoost conventional radiomics model were respectively, 0.861 (95% CI 0.807-0.915) and 0.818 (95% CI 0.717-0.918) for training (**Figure 2A**) and validation sets (**Figure 2B**), the highest out of the 7 models. Similarly, AUC values for the XGBoost DLR model were 0.913 (95% CI 0.872-0.954) for training (**Figure 2C**, **Table 4**) and 0.832 (95% CI 0.735-0.929) for the validation set (**Figure 2D**, **Table 4**). Therefore, compared to conventional radiomics, DLR was more accurate for predicting LNM in LSCC, with XGBoost DLR being the most optimal.

**Table 4:** Receiver operating characteristic curve (ROC) analyses of 7 deep learning radiomics (DLR) models for training and validation sets.

Model	Accuracy	AUC (95% CI)	Sensitivity	Specificity	PPV	NPV
Training dataset						
SVM	0.829	0.89 (0.8407-0.9399)	0.866	0.804	0.753	0.897
ExtraTrees	0.787	0.892 (0.8458-0.9388)	0.851	0.742	0.695	0.878
XGBoost	0.823	0.913 (0.8721-0.9544)	0.821	0.825	0.764	0.87
Light GBM	0.805	0.878 (0.8271-0.9279)	0.866	0.763	0.716	0.892
MLP	0.799	0.876 (0.8253-0.9276)	0.821	0.784	0.724	0.864
LR	0.823	0.90 (0.8548-0.9454)	0.881	0.784	0.737	0.905
RF	0.841	0.894 (0.8453-0.9419)	0.881	0.814	0.766	0.908
Validation dataset						
SVM	0.704	0.776 (0.6659-0.8863)	0.778	0.659	0.583	0.829
ExtraTrees	0.718	0.829 (0.7347-0.9235)	0.926	0.591	0.581	0.929
XGBoost	0.746	0.832 (0.7351-0.9290)	0.815	0.705	0.629	0.861
Light GBM	0.789	0.815 (0.7120-0.9184)	0.704	0.841	0.731	0.822
MLP	0.789	0.859 (0.7743-0.9446)	0.741	0.818	0.714	0.837
LR	0.69	0.783 (0.6729-0.8927)	0.778	0.636	0.568	0.824
RF	0.69	0.79 (0.6846-0.8945)	0.852	0.591	0.561	0.867

AUC, area under the curve; SVM, support vector machine; GBM, gradient boosting machine; MLP, multilayer perceptron; LR, logistic regression; RF, random forest; PPV, positive predictive value; NPV, negative predictive value.

### 3.3 Nomogram model combining clinical imaging characteristics and XGBoost DLR was the most optimal for predicting LNM in LSCC

**Figure 3:**
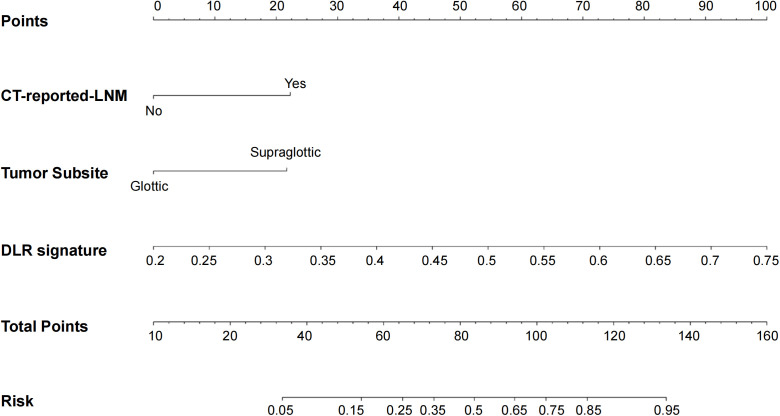
Predictive nomogram for LNM in LSCC patients, obtained by combining the clinical features of LNM being reported from computed tomography (CT) images, as well as whether the primary LSCC tumor is located in the glottis or supra-glottis, with XGBoost DLR features.

**Figure 4:**
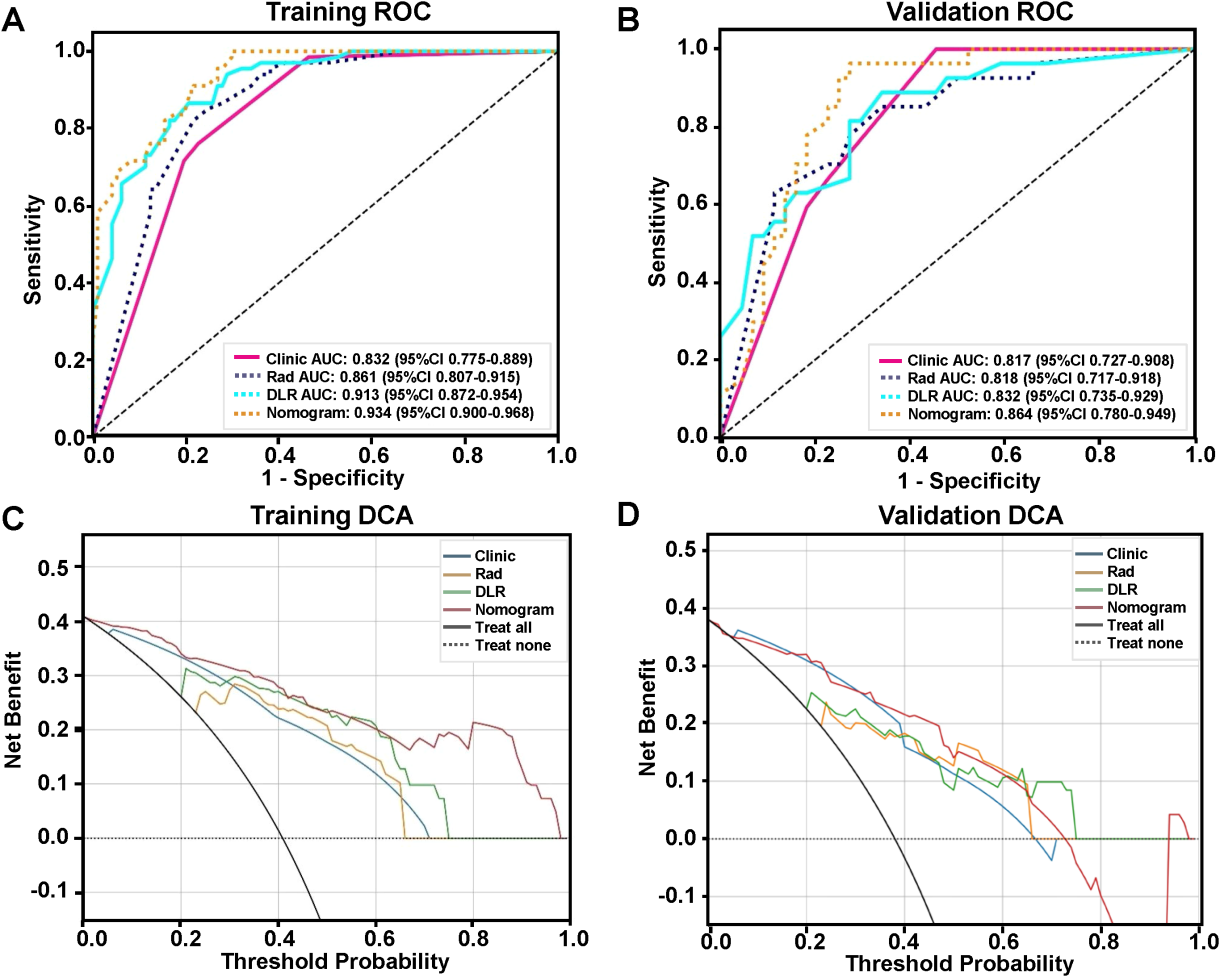
Comparing the accuracy and clinical utility of clinical features, conventional radiomics, DLR, and nomogram models for predicting LNM in LSCC patients. ROC curves for the 4 models on **(A)** training and **(B)** validation sets. Decision curve analysis (DCA) of the 4 models for **(C)** training and **(D)** validation sets.

After examining clinical features, conventional radiomics, and DLR models, we established the predictive nomogram by combining the clinical features model with the XGBoost DLR model (**Figure 3**). The nomogram, compared to the other 3 models, had the highest AUC values in ROC curve analysis, at, respectively, 0.934 (95% CI 0.900-0.968) for training, and 0.864 (95% CI 0.780-0.949) for the validation sets (**Table 5**, **Figures 4A, B**). Furthermore, the nomogram had the greatest clinical utility under DCA analysis, for both training (**Figure 4C**) and validation sets (**Figure 4D**), being significantly greater than assuming that all LSCC had LNM, or none did.

**Table 5:** ROC curve analyses for clinical imaging features, conventional radiomics, DLR and nomogram models, among training and validation sets.

Model	Accuracy	AUC (95% CI)	Sensitivity	Specificity	PPV	NPV
Training dataset						
Clinical Imaging Features	0.768	0.832 (0.7753-0.8890)	0.716	0.804	0.716	0.804
Conventional Radiomics	0.774	0.861(0.8065-0.9154)	0.642	0.866	0.768	0.778
Deep Learning Radiomics	0.823	0.914 (0.8721-0.9544)	0.821	0.825	0.764	0.87
Nomogram	0.817	0.934 (0.9004-0.9680)	0.985	0.701	0.695	0.986
Validation dataset						
Clinical Imaging Features	0.732	0.817 (0.7270-0.9077)	0.667	0.773	0.643	0.791
Conventional Radiomics	0.775	0.818 (0.7172-0.9183)	0.593	0.886	0.762	0.78
Deep Learning Radiomics	0.746	0.832 (0.7351-0.9290)	0.815	0.705	0.629	0.861
Nomogram	0.803	0.864 (0.7795-0.9494)	0.926	0.727	0.676	0.941

## 4 Discussion

In this study, we constructed a predictive nomogram for LNM in LSCC patients, which combined DLR features extracted from preoperative enhanced CT images with LNM-associated clinical imaging features from CT images, as well as whether the primary tumor was in the glottis or supra-glottis. This model was significantly more accurate than either clinical imaging features, conventional radiomics, or DLR models alone. Additionally, combining deep learning features with conventional radiomics in the DLR model significantly increased its accuracy versus that of conventional radiomics. Therefore, the nomogram was able to accurately predict LNM in LSCC, based on pre-operative CT scans, potentially serving as a valuable tool for identifying appropriate surgical strategies and improving patient outcomes.

Current approaches to evaluate LNM statuses in LSCC patients are traditional imaging methods, such as ultrasound and CT, but they have limited capabilities for examining LN<10 mm and distinguishing infected from metastatic LN. Nevertheless, some previous studies have shown that risk factors for LNM in LSCC include primary tumor location and size, the latter represented as the T-value in the TNM staging system, along with tumor differentiation extents and spread in preoperative CT reports (17, 18). Indeed, our study confirmed that primary tumor sites and preoperative CT reports of LNM were significant LNM risk factors. However, risk factors solely identified by clinical imaging are disfavored as indicators for conducting radical surgery, as they could result in excessive LN dissection and postoperative complications (19). As a result, radiomics became an attractive alternative for predicting LNM in LSCC, thus providing more accurate LSCC diagnoses and prognoses that could aid in devising effective treatment strategies (8, 9). In fact, their predictive capabilities for LNM in LSCC have already been demonstrated by Zhao et al. (12), who devised a radiomics-based predictive nomogram for LNM in LSCC, by extracting radiomics features from laryngeal cancer CT images, followed by combining them with CT-reported LN statuses and independent clinical risk factors. Similarly, Zhang et al. was able to predict LNM in head and neck SCC with their nomogram, which was developed by combining iodine-based radiomics imaging features, histological grading, and LN status reported in CT (20). All these nomograms thus demonstrate that radiomics-based models, involving imaging features extracted from primary lesions, are highly accurate for predicting LNM. This was in accordance with the results from this study, in which imaging features from both conventional and DLR machine learning algorithms were highly accurate for predicting LNM in LSCC.

However, it should be noted that obtaining tumor imaging features, whether derived from radiomics or the CT images directly, requires manually delineating the tumor areas, which is a time-consuming task with substantial subjectivity. Furthermore, manually identifying imaging features are restricted by human limitations in distinguishing fine image features, thereby posing significant difficulties for developing imaging-based predictive models. Such difficulties, though, could be mitigated by deep learning methods, which are able to extract high-level features from images in a data-driven manner (21, 22). Deep learning requires images to be pre-processed into sections containing the largest cross-section of the tumor, which can be automated, while conventional radiomics requires manually delineating the tumor regions (23, 24). Indeed, for deep learning feature extraction, a fixed size bounding box, covering the entire tumor region, is used, which is able to provide information from both within and the surrounding vicinity of the tumor (25). The combination of this information with conventional radiomics features has been found in previous studies to improve the accuracy of the resulting DLR models (10, 20). However, few studies have examined whether such a combination could also accurately predict LNM in LSCC, which we addressed in this study. We incorporated both conventional radiomics and deep learning features into different machine-learning models. The most accurate machine-learning model was then combined with clinical imaging features to form a predictive nomogram. This nomogram was highly accurate for predicting LNM in LSCC under ROC, as well as having greater clinical utility under DCA than clinical features, conventional radiomics, or DLR features alone. In fact, the addition of deep learning features to improve predictive accuracy was supported by findings from Wang et al., who observed that combining 2D and 3D deep learning features, along with radiomics and clinical characteristics, was more accurate for identifying occult LNM in LSCC than any of those features alone (26). Additionally, Liao et al. found that a deep-learning-based model was more accurate for predicting LSCC survival than TNM staging (27). Therefore, DLR incorporation into nomograms could further improve their LNM prognostication accuracies.

There are a number of limitations in this study, one of which is its retrospective nature, which, along with the small sample size of 235 patients, particularly with the training set, could result in an “overfitting” of the predictive nomogram, rendering it less accurate in identifying LNM. Therefore, future studies, with larger sample sizes and multiple centers, as well as scale- or meta-learning approaches, are needed to verify the predictive capabilities of the nomogram. Another limitation is that our study focused more on the characteristics of the primary tumor, and less on LN, which is due to positive LNM not being consistently visible under CT examination, making it difficult to match with LN biopsy results, especially if multiple LN are involved. Additionally, tumor segmentation was performed manually, which is dependent on the experience of the individual radiologist, leading to the nomogram results being too subjective for clinical applications. Consequently, to increase the nomogram reliability, future investigations should apply automatic segmentation methods and focus on LN results, which could provide more objective predictive assessments for LNM in LSCC.

## 5 Conclusion

In summary, we developed a predictive nomogram for LNM in LSCC, based on DLR features extracted from pre-operative CT, combined with the following clinical imaging features: LNM being present in CT images and whether the primary tumor was in the glottis or supra-glottis. This nomogram, compared to clinical imaging features, conventional radiomics, or DLR alone, was significantly more accurate for predicting LNM occurrence in LSCC patients, thereby providing an efficient, non-invasive method for preoperatively predicting LNM, which could greatly assist in devising individualized treatment strategies for LSCC.

## Data Availability

The raw data supporting the conclusions of this article will be made available by the authors, without undue reservation.
