# Supplementary material for: Deep learning radiomics nomogram predicts lymph node metastasis in laryngeal squamous cell carcinoma
Source: Front Oncol. 2025 Aug 12;15:1573687. doi: 10.3389/fonc.2025.1573687 (PMC12378036; doi:10.3389/fonc.2025.1573687)
Supplement: Supplementary file 1 [file Presentation1.pptx]

## Slide 1
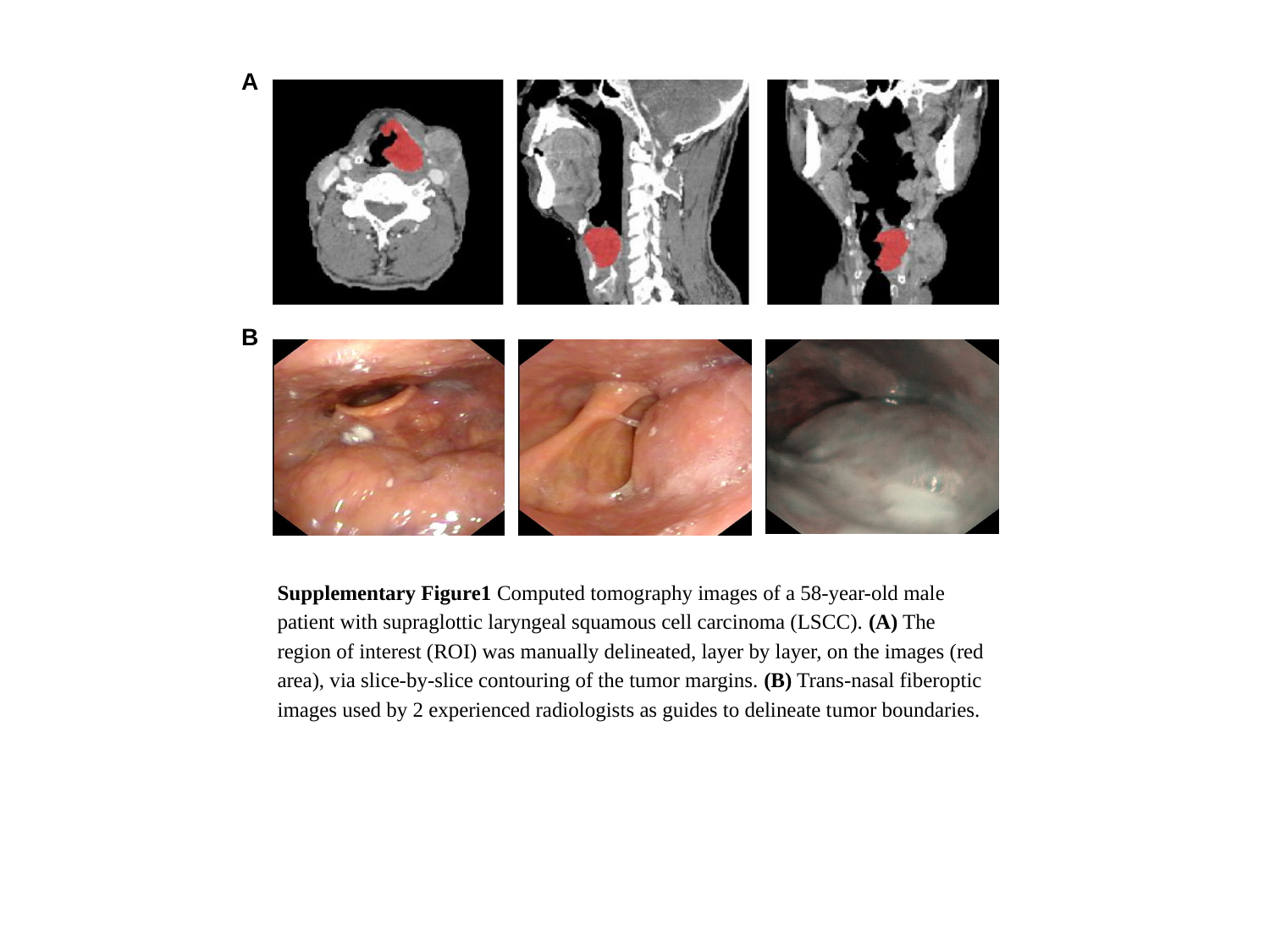

A
B
Supplementary Figure1 Computed tomography images of a 58-year-old male patient with supraglottic laryngeal squamous cell carcinoma (LSCC). (A) The region of interest (ROI) was manually delineated, layer by layer, on the images (red area), via slice-by-slice contouring of the tumor margins. (B) Trans-nasal fiberoptic images used by 2 experienced radiologists as guides to delineate tumor boundaries.
