# Supplementary figures and images for: Deep learning radiomics nomogram predicts lymph node metastasis in laryngeal squamous cell carcinoma
Source: Front Oncol. 2025 Aug 12;15:1573687. doi: 10.3389/fonc.2025.1573687 (PMC12378036; doi:10.3389/fonc.2025.1573687)

## Slide 1
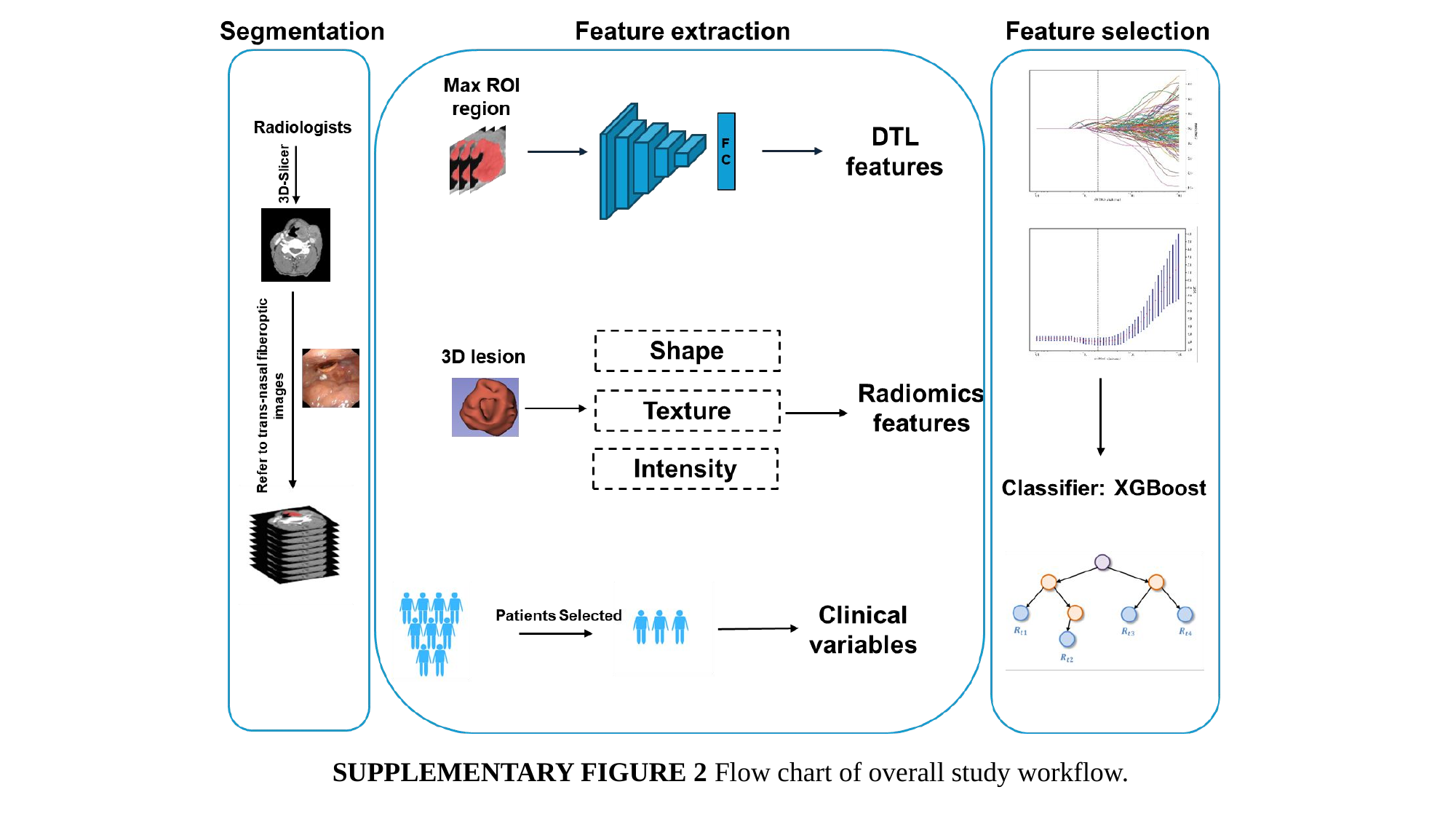

SUPPLEMENTARY FIGURE 2 Flow chart of overall study workflow.

Supplement: Supplementary file 2 [file Presentation2.pptx]
