# Supplementary material for: Deep learning radiomics nomogram predicts lymph node metastasis in laryngeal squamous cell carcinoma
Source: Front Oncol. 2025 Aug 12;15:1573687. doi: 10.3389/fonc.2025.1573687 (PMC12378036; doi:10.3389/fonc.2025.1573687)
Supplement: Supplementary file 3 [file Presentation3.pptx]

## Slide 1
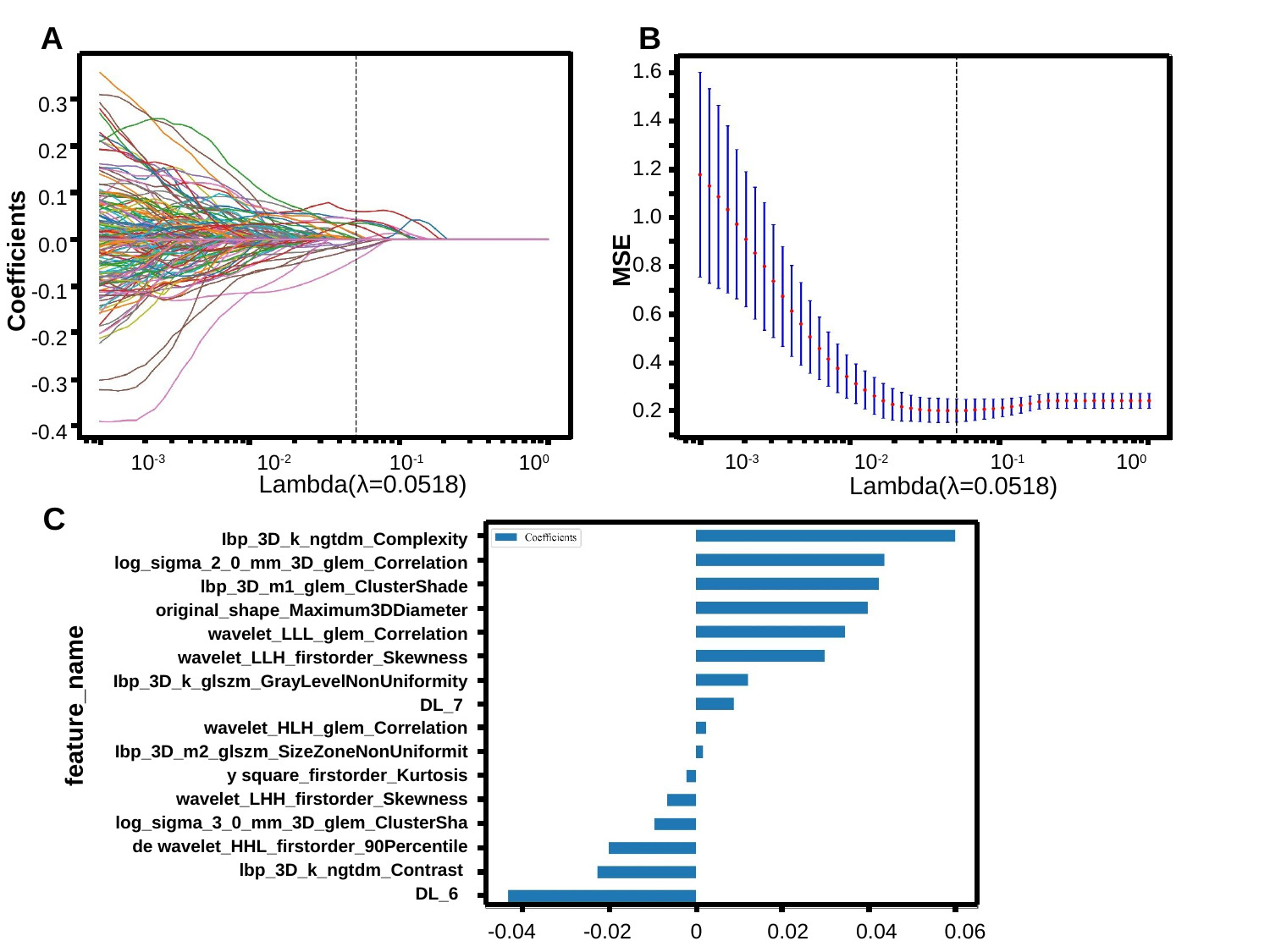

A
B
1.6
1.4
1.2
1.0
0.8
0.6
0.4
0.2
0.3
0.2
0.1
0.0
-0.1
-0.2
-0.3
-0.4
Coefficients
MSE
 10-3 10-2 10-1 100
 10-3 10-2 10-1 100
Lambda(λ=0.0518)
Lambda(λ=0.0518)
C
Ibp_3D_k_ngtdm_Complexity
log_sigma_2_0_mm_3D_glem_Correlation
lbp_3D_m1_glem_ClusterShade original_shape_Maximum3DDiameter wavelet_LLL_glem_Correlation wavelet_LLH_firstorder_Skewness Ibp_3D_k_glszm_GrayLevelNonUniformity
 DL_7
wavelet_HLH_glem_Correlation Ibp_3D_m2_glszm_SizeZoneNonUniformity square_firstorder_Kurtosis wavelet_LHH_firstorder_Skewness log_sigma_3_0_mm_3D_glem_ClusterShade wavelet_HHL_firstorder_90Percentile lbp_3D_k_ngtdm_Contrast
DL_6
feature_name
-0.04 -0.02 0 0.02 0.04 0.06

## Slide 2
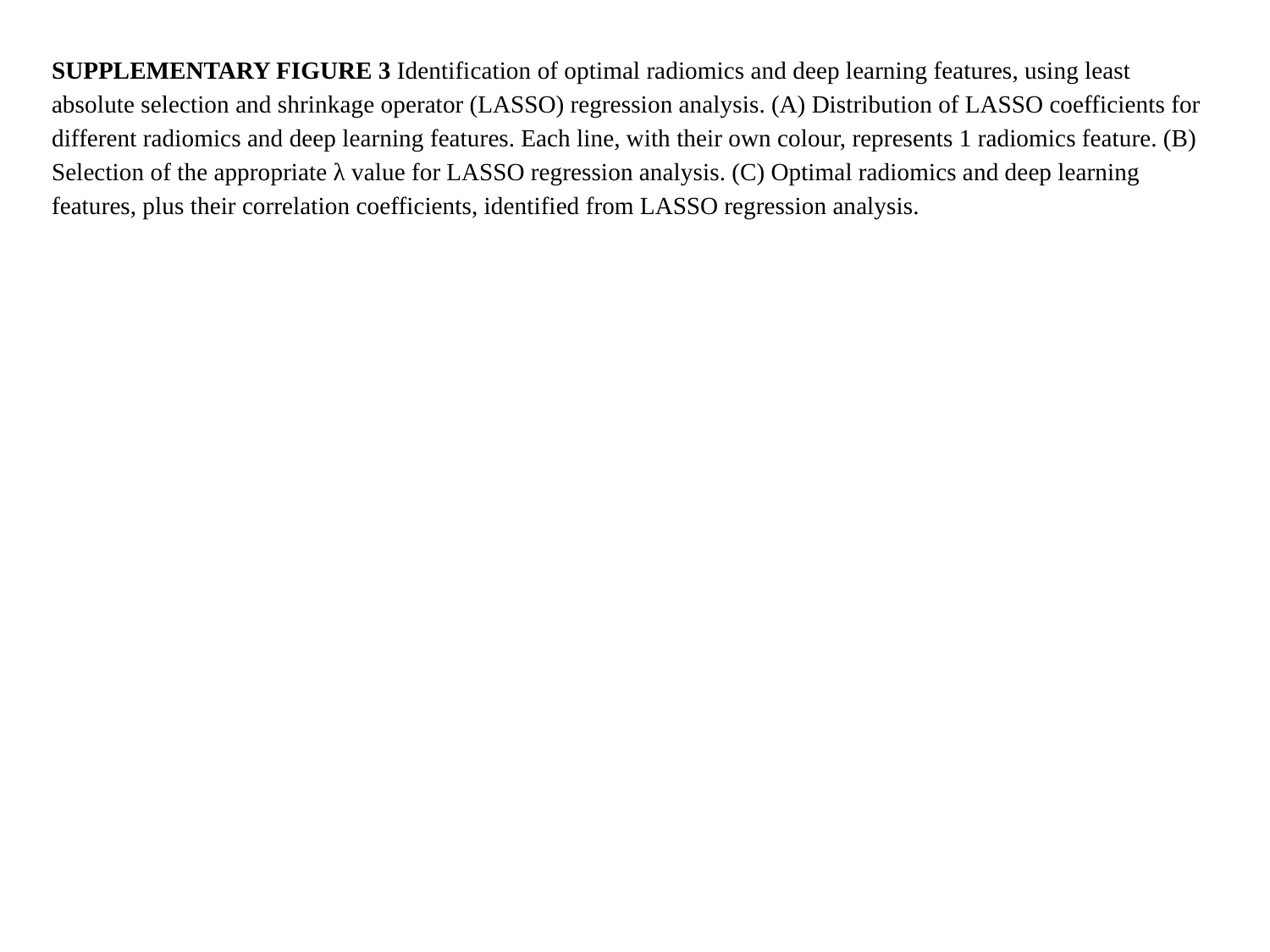

SUPPLEMENTARY FIGURE 3 Identification of optimal radiomics and deep learning features, using least absolute selection and shrinkage operator (LASSO) regression analysis. (A) Distribution of LASSO coefficients for different radiomics and deep learning features. Each line, with their own colour, represents 1 radiomics feature. (B) Selection of the appropriate λ value for LASSO regression analysis. (C) Optimal radiomics and deep learning features, plus their correlation coefficients, identified from LASSO regression analysis.
